# Antifungal susceptibility pattern and biofilm-related genes expression in planktonic and biofilm cells of *Candida parapsilosis* species complex 

**DOI:** 10.18502/cmm.5.4.1950

**Published:** 2019

**Authors:** Mona Modiri, Seyed Jamal Hashemi, Roshanak Daie GhazvinI, Sadegh Khodavaisy, Ali Ahmadi, Mansoureh Ghaffari, Sassan Rezaie

**Affiliations:** 1 Department of Medical Parasitology and Mycology, School of Public Health, Tehran University of Medical Sciences, Tehran, Iran; 2 Department of Microbiology, Faculty of Science, Islamic Azad University, Varamin-Pishva, Iran

**Keywords:** Antifungal susceptibility, Candida parapsilosis complex, Gene expression

## Abstract

**Background and Purpose:**

*Candida parapsilosis* complex isolates are mainly responsible for nosocomial catheter-related infection in immunocompromised patients. Biofilm formation is regarded as one of the most pertinent key virulence factors in the development of these emerging infections. The present study aimed to compare *in vitro* antifungal susceptibility patterns and biofilm-related genes expression ratio in planktonic and biofilm’s cells of clinically *C. parapsilosis* complex isolates.

**Materials and Methods:**

The current study was conducted on a number of 17 clinical *C. parapsilosis *complex (10 *C. parapsilosis sensu stricto*, 5 *C. orthopsilosis*, and 2* C. metapsilosis*). The antifungal susceptibility patterns of amphotericin B, ﬂuconazole, itraconazole, voriconazole, posaconazole, and caspofungin in planktonic and biofilm forms were closely examined using CLSI M27-A3 broth microdilution method. The expression levels of biofilm-related genes (*BCR1, EFG1*, and *FKS1*) were evaluated in planktonic and biofilm’s cells using Real-time polymerase chain reaction (PCR) technique.

**Results:**

The obtained results indicated that all *C. parapsilosis *complex isolates were able to produce high and moderate amounts of biofilm forms. In addition, the sessile minimum inhibitory concentrations were reported to be high for fluconazole (≥ 64 µg/ml), itraconazole, voriconazole, and posaconazole (≥ 16 µg/ml), as compared to planktonic minimum inhibitory concentrations. Moreover, a significant difference was observed between antifungal susceptibility patterns for all azole antifungal agents (*P<0.05*). Furthermore, the *BCR1* overexpression was considered significant in biofilms with regard to planktonic cells in *C. parapsilosis* species complex (*P=0.002*).

**Conclusion:**

*C. parapsilosis* complex isolates were found susceptible to most of the tested antifungal drugs, while biofilms demonstrated a noticeable resistant to azoles. The marked discrepancy noted in antifungal susceptibility patterns among these species should be highlighted to achieve effective therapeutic treatment.

## Introduction

*Candida parapsilosis* is one of the main commensal species of genus *Candida* which is isolated from other sources, such as hospital environments, soil, and domestic animals, contrary to other human pathogens of *Candida* species [1]. *C. parapsilosis* is considered one of the leading causes of catheter-related infections in hospitalized patients, particularly in immunocompromised individuals and neonates. This can be attributed to its prominent ability to form biofilms on indwelling catheters and other medical and prosthetic devices, as well as nosocomial transmission by hand carriage [2]. *C. parapsilosis* was reclassified into three newly-discovered species, namely* C. parapsilosis sensu stricto, C. orthopsilosis*, and *C. metapsilosis *[3]. These species *cannot* be *phenotypically differentiated* in the sense that they are not identifiable by conventional methods [4]. In addition, they are different in their pathogenicity and antifungal susceptibility profiles [5]. Biofilm formation is regarded as one of the major virulence attributes resulting in antifungal resistance and host immune system protection. These structures possibly increase the persistence of yeast infection owing to colonization on biotic, as well as abiotic surfaces, such as venous catheters, intracardiac prosthetic devices, and other implanted devices [6]. Therefore, the investigation of different aspects and mechanisms of biofilm formation involves the application of various methods [7, 8]. Moreover, bioﬁlm development by *Candida* species is a complicated process adjusted through well-coordinated regulatory network genes as core components of persistent infection [9]. Biofilm and cell wall regulator 1 (*BCR1*), Beta-1, 3-glucan synthase catalytic subunit 1 (*FKS1*), and Enhanced filamentous growth protein 1 (*EFG1*) are referred to as biofilm-related genes in *C. albicans *and* C. parapsilosis *[10]. *BCR1 *as the main transcription factor plays an essential role in the early adhesion stage of biofilm formation in *C. albicans* and *C. Parapsilosis* [11]. On the other hand, the *EFG1* transcription factor is required for bioﬁlm formation and hyphal growth in *C. parapsilosis* [12]. Although members of *C. parapsilosis *complex are usually susceptible to azole antifungals, resistance has been reported. Few studies exist in Iran on biofilm antifungal susceptibility characteristics and *C. parapsilosis* species complex regulatory network gene. The present study compared *in vitro* antifungal susceptibility and the biofilm-related genes expression ratio in planktonic cells and biofilms among clinical *C. parapsilosis* complex isolates.

## Materials and Methods

***Fungal isolates***

The analysis was performed on a panel of 17 clinical isolates of *C. parapsilosis* complex. *C. parapsilosis* *sensu stricto* (n=10) and *C. orthopsilosis* (n=5), were obtained from Tehran Medical Mycology Laboratory (TMML) collection, Tehran, Iran and *C. metapsilosis* (n=2) were provided by Canisius-Wilhelmina Ziekenhuis (CWZ), Nijmegen, the Netherlands. In addition, clinical strains were sourced from blood, sputum, Broncho-alveolar lavage (BAL), nails, and vaginal discharge samples. All the isolates were initially identified by matrix-assisted laser desorption ionization-time of flight mass spectrometry (MALDI-TOF) and confirmed by sequencing of internal transcribed spacer ribosomal DNA region [13, 14]. 

***Biofilm formation***

Biofilm formation protocol was adapted from that of Pierce *et al.* [15] with modifications. In brief, Sabouraud Dextrose Agar (SDA, Difco) was used for the initial cultivation of all isolates at 37°C for 48 h. Thereafter, the cells were inoculated in Sabouraud dextrose broth (SDB, Difco) and incubated at 37°C for 18-24 h. The cells were then harvested by centrifugation at 3000×g and were washed twice in sterile phosphate-buffered saline (PBS, pH=7.4). They were suspended in about 10-15mL of RPMI 1640 medium (Sigma-Aldrich, St. Louis, USA) buffered to pH 7.0 with 0.165 M-morpholinepropanesulfonic acid (MOPS; Sigma-Aldrich). The cellular density was adjusted to approximately 1×10^6^ CFU/ml (OD600 = 1.0). Thereafter, 100µL of suspension was transferred into 96-well microtiter plates (Suzhou Conrem Biomedical Technology Co., Ltd, China) and incubated at 37ºC for 48 h. 

***Biofilm quantification***

Quantification of biofilm formation by clinical isolates was performed using Crystal violet (Merck, Germany) staining method (CV), according to the protocol described by Silva *et al.* [16]. In a nutshell, following biofilm formation, the wells were washed with PBS, methanol was added to each well, and CV (1% v/v) was then added to wells succeeded by acetic acid (33% v/v). The absorbance was measured at 570nm. Isolates were classified into high, moderate, and low biofilm producers, according to the study conducted by Stepanovic *et al.* [17]. 

***Antifungal susceptibility testing in Planktonic cells***

*In vitro *antifungal susceptibility testing against planktonic cells was carried out using CLSI M27-A3 broth microdilution method [18]. All the isolates were exposed to six antifungal drugs, including amphotericin B (AMB, Bristol-Myers-Squibb, Woerden, The Netherlands), fluconazole (FLU, Pfizer Central Research, Sandwich, UK), itraconazole (ITC, Janssen Research Foundation, Beerse, Belgium), voriconazole (VRC, Pﬁzer, New York, NY, USA), posaconazole (PSC, Merck, Whitehouse Station, NJ), and caspofungin (CAS; Pfizer). Apart from 0.063-64 μg/ml for FLU and 0.008- 8 μg/ml for CAS, a final concentration of 0.016-16 μg/ml were used for AMB, ITC, VRC, and PSC. All identified yeasts were sub-cultured on SDA plates at 35 °C for 24 h. Inoculum suspensions were prepared and adjusted to the transmission of 75%-77% at 530 nm (approximate 1×10^6^–5×10^6 ^CFU/ml). The inoculum suspensions were diluted 1: 1000 in RPMI 1640 medium and the final inoculum in wells was within 0.5×10^3^-2.5×10^3^ CFU/ml. The microdilution plates were incubated at 35 °C. After 24 h, the minimum inhibitory concentration (MIC) endpoints were determined using a reading mirror and were defined as the lowest concentration of drugs that significantly reduced growth (>50%), as compared to the growth of a drug-free control. However, the MIC for AMB was defined as the lowest concentration at which there was 100% inhibition of growth. MIC_50_ and MIC_90_ were defined as minimum inhibitory concentrations required to inhibit the growth of 50% and 90% of organisms. *C. parapsilosis* (ATCC 22019) and *C. krusei* (ATCC 6258) standard strains were used as quality control. Due to the absence of CLSI clinical breakpoints values (CBPs) for AMB, ITC, and PSC, their corresponding MIC values were interpreted based on epidemiological cut-off values (ECV) and non-wild type (NWT) values when the MIC values were >2, >0.5 and >0.25 μg/ml, respectively. The new CBPs were used for FLU (≤ 2 μg/ml susceptible (S), 4 μg/ml susceptible dose-dependent (SDD), and ≥ 8μg/ml resistant (R), VRC (≤ 0.125 μg/ml S, 0.25-0.5 μg/ml intermediate (I) and ≥1 μg/ml R) and CAS (≤ 2 μg/ml S, 4 μg/ml I and ≥8 μg/ml R) [19-21].

**Table 1:** The specific primers for Real-Time Polymerase Chain Reaction

**Gene**	**Accession no.**	**Primer**	**Primer Sequence**	**PCR product length (bp)**
*BCR1*	KJ610856.1	BCR1-S1 BCR1-AS1	ACCACTACAGGGACAGCCAT AAGAATTGGCGTTACCGGCG	248
*EFG1*	HE605209.1	EFG1-S EFG1-AS1	AAGTCGAGACCCACCCATTG TTGTGTCCCTTTGCACTGCC	201
*FKS1*	XM_003867859.1	FKS1-S1 FKS-AS1	TCATCACACACTTTCACGGCA TCGACAGCATACATCAATCCC	248
*ACT1*	XM_003869098.1	ACT1 – S1 ACT1 – AS1	ACGGTATTGTTTCCAACTGGGACG TGGAGCTTCGGTCAACAAAACTGG	110

***Antifungal susceptibility testing in sessile cells***

The aforementioned microtiter-based assay was utilized to determine the sessile minimum inhibitory concentrations (SMICs) [22]. The biofilms were washed with PBS following 48 h of bioﬁlm growth in 96-well microtiter plates as mentioned above. In addition, final concentration which were used included 0.03-16 μg/ml for AMB, ITC, VRC, and PSC, 0.5-64 μg/ml for FLU, and 0.03-8 μg/ml for CAS. Thereafter, 200µL of each drug concentration was added to the respective wells and the plates were incubated at 37°C for 48 h. Positive control wells contained biofilms without any drug. Thereafter, the biofilms were washed two times with sterile PBS and 3(4,5-Dimethylthiazol-2-yl)-2,5-diphenyl tetrazolium bromide (MTT) reduction method was used to determine metabolic activity using the assays as previously described by Mosmann *et al.* [23]. Biofilms were washed with sterile PBS 48 h after drug exposure and MTT solution (stock solution 5mg/ml suspended in PBS; Sigma) was added to each well. Plates were covered with aluminum foil and were incubated at 37ºC for 2 h. Dimethyl sulfoxide (DMSO, Merck) was then added and the absorbance of the solution was assessed spectrophotometrically at 570nm. The SMICs were described as the lowest drug concentrations at with 50% decrease in absorbance, as compared to drug-free growth control well. The isolates were tested in duplicate. 

***Gene’s expression analysis***

For the purpose of the current study, genes related to the production of biofilm (*BCR1, EFG1*) and matrix components of β-1, 3 glucan (*FKS1*) were selected and their expression was evaluated in all isolates before and after biofilm formation. Primers were designed using Primer 3 software (Table 1). 

***RNA extraction and cDNA synthesis***

Bioﬁlms were formed in 24-well microtiter plates and were incubated for 48 h as mentioned earlier, the wells were then washed with sterile PBS and the biofilms were scraped from the wells. To disintegrate the biofilm matrix, the solution was sonicated (UCE ultrasonic processor co, Ltd, China), and the cells were harvested using centrifugation at 3000×g [24]. Moreover, in planktonic form, all isolates were cultured on SDA medium at 37 ^o ^C for 48 h. Total RNAs were extracted both 48-h biofilms and planktonic cells by Trizol method as already noted [25]. In order to attain a product with good quality and purity, the ratio of optical density at 260nm and 280nm should be above 1.6. The cDNA from 1µg of total RNA was synthesized using 2x RT-PCR pre-mix Taq kit (Biofact, Korea), according to the manufacturer’s instructions. 

***Real-time polymerase chain reaction***  

Gene’s expression was assessed using BioFACT™ Real-Time PCR Series kit (Biofact, Korea), according to the manufacturer's protocol on a Rotor Gene Q device (Qiagen, Germany). The Real-Time PCR protocol was run as follows: initial denaturation at 95°C for 13 min followed by 45 cycles of denaturation (95°c, 20 secs), annealing (58°C, 20 secs), and extension (72°C, 30 secs), succeeded by a final extension step at 72°C for 1 min and melting step performed at 72-95 °C*. Act 1* as endogenous control (house-keeping gene) was used to normalize and confirm the PCR process. The expression ratios in biofilms were calculated by REST2009 Software (V2.0.13) using ΔΔCt method. 

***Statistical Analysis***

The biofilms quantifications were presented as OD values mean±standard deviation (SD). All the obtained data were analyzed in SPSS software (version 25). Student's t-tests were used to measure statistical differences between two or more groups. Differences between the SMIC values and their MICs were examined using Wilcoxon Signed Rank’s test. In addition, the association between expressions of biofilm-related genes and biofilm-forming phenotype was evaluated using the Pearson or Spearman’s Correlation coefficient (r). A *P-value* less than 0.05 was considered statistically signiﬁcant. 

## Results

***Biofilm quantification by crystal violet staining method***

**Figure 1:**
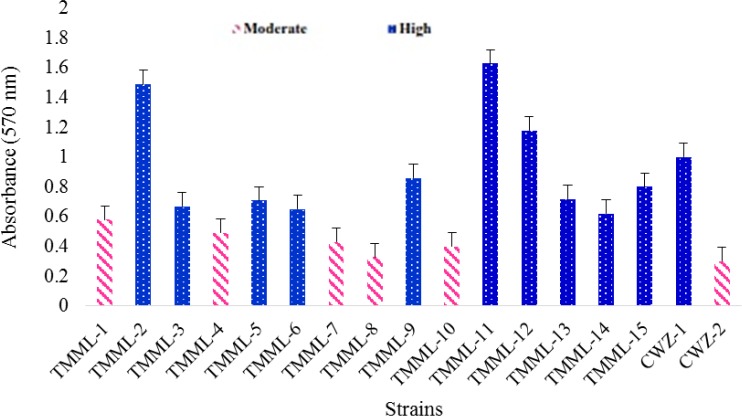
Bioﬁlm quantiﬁcation of *C. parapsilosis sensu stricto* (TMML-1 to TMML-10); *C. orthopsilosis* (TMML-11 to TMML-15); *C. metapsilosis* (CWZ-1 to CWZ-2) isolates using crystal violet staining method

Figure 1 indicates biofilm quantification by CV staining for *C. parapsilosis *complex isolates. All *C. orthopsilosis* and 50% of* C. parapsilosis sensu stricto*, and *C. metapsilosis* isolates formed high amounts of biofilms on the basis of CV staining assay (OD > 0.60). No statistically signiﬁcant difference was observed among *C. parapsilosis* species complex in terms of biofilm biomass production (*P=0.214*). 

***Planktonic and biofilm susceptibility testing***

The distribution of MICs for planktonic and bioﬁlm-grown *C. parapsilosis* complex isolates is depicted in Table 2. All isolates in planktonic forms were susceptible to VRC (MIC≤ 0.125 µg/ml), CAS (MIC ≤ 2 µg/ml), AMB (≤ 2 μg/ml) and PSC (≤ 0.25 μg/ml). All  

**Table 2:** Minimum inhibitory concentration (MICs) distribution of antifungal drugs for planktonic and sessile (bioﬁlm) cells of *Candida parapsilosis* species complex

**No. of isolates for which the MIC(µg/mL) was :**																
**Species (n)**	**Antifungal agents**	**Type of MIC**	**0.008**	**0.016**	**0.031**	**0.062**	**0.125**	**0.25**	**0.5**	**1**	**2**	**4**	**8**	**≥16**	**32**	**≥64**
*Candida parapsilosis* *Sensu stricto* (n=10)	AMB FLU ITC VRC PSC CAS	PMIC ^a^ SMIC ^b^ PMIC SMIC PMIC SMIC PMIC SMIC PMIC SMIC PMIC SMIC		4 7 9	1 6 3 1	1 1 1 2	1 2 4 2	3 3	1 4 4	6 1 3 1	4 1	1 1	1 1	10 10 10		10
*Candida orthopsilosis* (n=5)	AMB FLU ITC VRC PSC CAS	PMIC SMIC PMIC SMIC PMIC SMIC PMIC SMIC PMIC SMIC PMIC SMIC		2 2 3	1 2 3 1	2 2 2 1	1	1 1 2	2 3 2	1 2	4			5 5 5		5
*Candida metapsilosis* (n=2)	AMB FLU ITC VRC PSC CAS	PMIC SMIC PMIC SMIC PMIC SMIC PMIC SMIC PMIC SMIC PMIC SMIC		1 2 2	1 1	1	1	1 1	1		2 2			2 2 2		2

^a ^PMIC: planktonic Minimum inhibitory concentration

^b ^SMIC: sessile Minimum inhibitory concentration

AMB; amphotericin B, FLU; fluconazole, ITC; itraconazole, VRC; voriconazole, PSC; posaconazole, CAS; caspofungin

*C. parapsilosis* complex isolates were susceptible to FLU, except for one resistant *C. parapsilosis sensu stricto* isolate (MIC=8 μg/ml). The one* C. parapsilosis sensu stricto*, two *C. orthopsilosis, *and two *C. metapsilosis* isolates had an non wild type (NWT) phenotype against ITC (> 0.5 μg/ml). The SMICs of biofilms were reported to be high for FLU (SMIC > 64µg/ml), ITC, VRC and PSC (SMIC > 16µg/ml), in comparison with their MICs planktonic forms. In addition, a significance difference was observed in SMICs for all azole antifungal agents, as compared to their planktonic MICs (*P < 0.05*). Only one *C.* *parapsilosis sensu stricto* isolate was found to be resistant to CAS (SMIC=8µg/ml) and had an NWT phenotype against AMB (> 2 µg/ml). However, no statistically significant difference was observed among the *C. parapsilosis* complex isolates in terms of the SMIC values for AMB (*P= 0.08*) and CAS (*P= 0.31*), in comparison with their planktonic MICs.

***Expression analysis***

**Figure 2:**
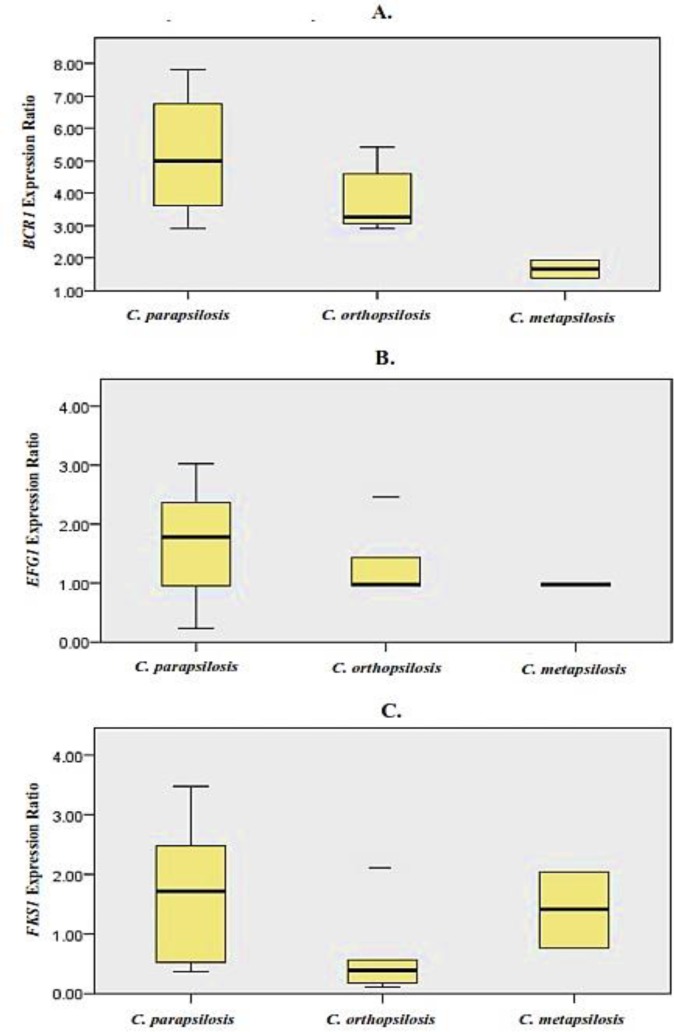
The expression ratio of A. *BCR1* gene, B. *EFG1* gene, C. *FKS1* gene in *C. parapsilosis *species complex. Relative gene expression is the ratio of expression under biofilm form relative to planktonic form. Values between 0 and 1 indicate low expression, while values >1 represent overexpression. The overexpression of *BCR1* was significant (*P=0.002*), while no significant overexpression was observed for *EFG1* (*P=0.17*) and *FKS1* (*P=0.22*)

Figure 2 demonstrates the expressions ratio of *BCR1, EFG1*, and *FKS1* genes in biofilms of *C. parapsilosis* complex isolates with respect to planktonic cells. A signiﬁcant overexpression of *BCR1* gene was detected in biofilms of all *C. parapsilosis* complex isolates (*P=0.002*). The highest expression variations for *BCR1* gene were noticed in biofilms of *C. parapsilosis sensu stricto *isolates (2.90-7.81-fold). On the other hand, *EFG1* and *FKS1* genes were not coordinately expressed in all *C. parapsilosis* complex isolates. The *EFG1* gene was upregulated only in biofilms of six and two isolates of *C. parapsilosis sensu stricto *and* C. orthopsilosis *(1.42 to 3.02-fold). The overexpression of *FKS1* gene were detected in biofilms of 6, 1 and 1 isolates of *C. parapsilosis sensu stricto, C. orthopsilosis *and* C. metapsilosis *(1.59 to 3.47-fold). In addition, no significant difference was noted between the expression of *EFG1* (*P=0.17*) and *FKS1* (*P=0.22*) genes in biofilms of *C. parapsilosis* complex isolates, relative to the planktonic cells. Moreover, the lack of correlation was demonstrated between expressions of biofilm-related genes and bioﬁlm forming phenotypes (high and moderate phenotypes; r = 0, *P = 0.02*).

## Discussion

There is a notable increase in the frequency of non-*C. albicans Candida* species, such as *C. parapsilosis,* despite the prevalence of *C. albicans* as the most common pathogen in infections [26, 27]. Since 2005 when *C. parapsilosis *complex was reclassified into three distinct species, several countries began to conduct surveillance studies on different characteristics of these species [28]. In the current study, a total of 10 C*. parapsilosis sensu stricto*, 5 *C. orthopsilosis* and 2 *C. metapsilosis* isolates were identified by sequencing of the internal transcribed spacer ribosomal DNA region. The high prevalence of *C. parapsilosis sensu stricto *reported in this research was consistent with previous studies conducted in Italy, Spain, Latin America, Turkey, Iran, and other Asian countries [14, 29-33]. Several countries reported a higher prevalence for *C. metapsilosis*, as compared to *C. orthopsilosis *[34-36]. A study carried out in India, indicated the highest prevalence of *C. orthopsilosis* (40.2%), in comparison with previous literature [37]. The rare isolation of *C. metapsilosis* is not yet clear in many studies; however, *C. metapsilosis* appears less virulent than other species within complex [5]. The current study compared antifungal susceptibility profiles of *C. parapsilosis* complex isolates grown as bioﬁlm and planktonic cells. All* C.* *parapsilosis* *sensu stricto* isolates were susceptible to all evaluated antifungal drugs except for one FLU resistant isolate and another isolate with ITC-NWT phenotype. In addition, none of the *C. orthopsilosis* and *C. metapsilosis* isolates were resistant to AMB, FLU, VRC, PSC and CAS tested antifungal agents, which is comparable to the results of previous studies performed inTurkey, Italy, Spain, Brazil and other Asian countries [29, 32, 33, 36, 38]. A recent study conducted by Maria *et al*. [37] in India indicated 16% FLU resistant isolates of *C. parapsilosis sensu stricto* which is contrary to the low levels of resistance reported in our study and previous literature [39, 40]. In addition, Rizzato *et al.*

[41] demonstrated the high resistance to FLU in 40% of *C. orthopsilosis* isolates. Moreover, based on the results of the study conducted by Salarci *et al.* [42] in Turkey, CAS resistance was observed in 14 *C. parapsilosis *isolates. In the present study, low levels of ITC resistance was detected in *C. parapsilosis* species complex, which is in line with the results of the studies performed by Canton *et al.* [29] and Ruiz et al. [43] . Resistance to antifungal drugs in *Candida* biofilm which is a commonly observed phenomenon presents daunting challenges to clinical treatments. Such a phenomenon may foster persistence in many catheter-related infections and lead to ineffective antimicrobial therapy [6]. High azole SMICs were observed for all of the tested isolates which indicated resistance to FLU, ITC, VRC, and PSC. The results of the current study were in agreement with several studies suggesting that azoles are not active against *C. albicans* and *C. parapsilosis* complex biofilms [44, 45]. In the same vein as previous findings, *C. parapsilosis* complex isolates demonstrated the biofilm susceptibility to AMB and echinocandins [45, 46]. Biofilm formation is a complex biological process under the control of the inherent genetic mechanisms of organisms [9]. The expression levels of three biofilm-related genes, namely *BCR1*, *EFG1*, and *FKS1*, were investigated in biofilms of seventeen *C. parapsilosis* complex isolates. Out of these three biofilm-related genes, *BCR1* was signiﬁcantly upregulated in biofilms of all *C. parapsilosis *complex isolates relative to the planktonic cells which revealed that this gene might be responsible for biofilm formation in *C. parapsilosis* species complex. On the same note, Nikoomanesh *et al*. [47] pointed out a positive relationship between expression of *BCR1* gene and biofilm formation in *C. albicans* isolates. Moreover, Pannanusorn *et al. *[46] suggested that bioﬁlm formation in *C. parapsilosis* isolates is both dependent and independent on* BCR1* gene. The results of the current study provide a remarkable insight into the antifungal susceptibility pattern and genes related to biofilm formation in *C. parapsilosis* species complex. Nonetheless, a serious limitation of this study was the small number of isolates belonging to the emerging identified species; therefore, the antifungal susceptibility pattern of *C. parapsilosis* species complex may not provide a true reflection of differences among these species. 

## Conclusion

The results of the present research were indicative of dramatic differences in antifungal susceptibility proﬁles of planktonic cells and biofilms among *C. parapsilosis* species complex, mainly with regard to azoles and even very little resistance should be taken into account to select effective antifungal therapy. The obtained ﬁndings highlighted that the *BCR1* gene might be responsible for biofilm development in *C. parapsilosis* species complex. Further investigation is highly recommended with a larger number of isolates to gain a better understanding of the distribution, susceptibility pattern, and virulence attributes of *C. parapsilosis* species complex in Iran. 
